# An innovative approach to control fish-borne zoonotic metacercarial infections in aquaculture by utilizing nanoparticles

**DOI:** 10.1038/s41598-024-74846-y

**Published:** 2024-10-25

**Authors:** Olfat A. Mahdy, Mai A. Salem, Mohamed Abdelsalam, Marwa M. Attia

**Affiliations:** 1 Parasitology Department, Faculty of Veterinary Medicine, Cairo University, Giza, 12211 Egypt; 2 Department of Aquatic Animal Medicine and Management, Faculty of Veterinary Medicine, Cairo University, Giza, Egypt

**Keywords:** Nanocontrol, *Clinostomum* spp., *Prohemistomum* sp., Chitosan, Silver, Selenium, FBZT, Biotechnology, Microbiology, Zoology

## Abstract

Fish-borne zoonotic trematodes (FBZTs) pose significant health risks and economic challenges worldwide. This study investigated the prevalence of encysted metacercariae (EMCs) in Nile tilapia (*Oreochromis niloticus*) from two Egyptian governorates and evaluated the antiparasitic efficacy of chitosan, silver, and selenium nanoparticles against these parasites. A cross-sectional analysis of 453 *O. niloticus* specimens revealed an overall EMC prevalence of 40.8%, with infection rates of 34.11% in Giza and 49.5% in Dakahlia. Clinostomid and Prohemistomid metacercariae were the most common, with mixed infections observed. Transmission electron microscopy characterized the synthesized nanoparticles, showing average diameters of 9.6–18.7 nm for chitosan, 13.2–19.8 nm for selenium, and 11.7–15.1 nm for silver nanoparticles. In vitro antiparasitic assays demonstrated varying efficacies among the nanoparticles. Against *Clinostomum* spp. metacercariae, chitosan nanoparticles showed the highest potency, achieving LC50 at 66 μg/ml after 30 min and LC90 at 100 μg/ml after 120 min. For *Prohemistomum vivax* EMCs, chitosan nanoparticles exhibited superior efficacy, achieving LC50 at 8 μg/ml after 1 h and LC90 at 16 μg/ml after 2 h. Silver and selenium nanoparticles showed lower efficacy for both parasite species. Scanning electron microscopy revealed significant ultrastructural damage to the parasite tegument following nanoparticle exposure, including disappearance of transverse ridges, integument shrinkage, and formation of blebs. This study provided valuable insights into the prevalence of FBZTs in Egyptian Nile tilapia and demonstrated the potential of nanoparticles, particularly chitosan, as effective antiparasitic agents. These findings pave the way for developing novel, targeted strategies to control fish-borne zoonotic trematodes, potentially reducing their impact on public health and aquaculture economies.

## Introduction

Fish-borne zoonotic trematodes (FBZTs) have emerged as a significant global health concern, garnering increased attention from researchers and public health officials worldwide^1,2^. The World Health Organization has reported that over 18 million people are infected with these parasitic flatworms, underscoring the magnitude of this often-overlooked health issue^3,4^. The major threat to the global spread of parasitic disorders caused by the causative agent helminthes is the large number of infected hosts, including humans^5^. Among the diverse families of digenetic trematodes, Clinostomidae and Prohemistomidae stand out due to their complex life cycles involving both molluscan and vertebrate hosts, presenting unique challenges for control and prevention. Clinostomid metacercariae (MCs) have been extensively documented in freshwater fish species across the globe, with their ability to encyst in muscle and other tissues as infective stages posing significant risks to both human health and aquaculture industries^6–8^**.** The life cycle of these parasites is intricate, with piscivorous birds serving as definitive hosts for adult flukes. These adult flukes produce eggs that are shed into waterways, perpetuating the parasite’s life cycle and complicating eradication efforts. The economic impact of heavy metacercarial infections in farmed fish cannot be overstated, as they lead to substantial losses and raise critical food safety concerns^9–11^. A significant challenge in addressing the FBZT issue lies in the morphological identification of clinostomid MCs. The limited distinguishing taxonomic features visible in cyst stages have hampered accurate diagnosis and species-level classification, necessitating the development of more advanced identification techniques^8,12^. This limitation has spurred researchers to explore innovative approaches for parasite detection and control.

Among the zoonotic flukes of particular concern is *Prohemistomum vivax*, which has demonstrated considerable health and economic impacts. The groundbreaking work of Nasser^13^ confirmed the zoonotic potential of *P. vivax* by documenting human infection in Cairo, Egypt. This parasite, known to inhabit the intestines of fish-eating birds and mammals, including humans, has been recorded across diverse geographical regions, including Egypt, Palestine, Japan, and Europe^14–16^. The widespread distribution of *P. vivax* underscores the need for effective control strategies that can be applied on a global scale^17,18^.

In recent years, the field of nanotechnology has opened up exciting new possibilities for addressing parasitic infections. The development of efficient and environmentally friendly methods to synthesize metal nanoparticles has gained considerable attention across various areas of nanotechnology. Among these, silver nanoparticles (AgNPs), chitosan nanoparticles (ChNPs), and selenium nanoparticles (SeNPs) have emerged as particularly promising due to their potential as antimicrobial agents and their wide applicability in biological and medical fields. Chitosan-silver-selenium nanocomposites (Ch-AgNPs) represent a cutting-edge group of bio-nanostructured hybrid materials. Their biocompatibility, biodegradability, and broad-spectrum antimicrobial and antifungal activities make them particularly attractive for biomedical applications^19^. This nanotechnology-based approach offers a cleaner, more cost-effective alternative to traditional mass treatment methods and heavy machinery, aligning with the growing demand for sustainable and environmentally friendly solutions in parasite control.

The potential of nanotechnology extends far beyond parasite control, with applications spanning nearly every aspect of life. From nanomaterial sciences and nanoelectronics to nanomedicine, the integration of nanotechnology into the chemical, physical, and biological world is rapidly advancing. As El Naschie^20^ astutely predicted, nanotechnology is poised to become a fundamental field of study for future generations, revolutionizing industries and scientific disciplines alike. Nano medicine, in particular, has emerged as a promising new research area in both human and veterinary science^21^. While metal nanoparticles have demonstrated high biocidal activity against bacteria, fungi, and viruses, their potential as antiparasitic agents remains relatively unexplored. This gap in knowledge presents an opportunity for ground-breaking research that could significantly impact the field of parasitology and public health. This study aimed to pioneer a novel methodology for controlling the larval stages of FBZTs using a range of basic nanoparticles, including chitosan, silver, and selenium. By characterizing these nanoparticles using transmission electron microscopy (TEM) and determining their LC50 and LC90 values, we sought to establish a foundation for future antiparasitic nanoparticle applications. Furthermore, this research was designed to assess the morphological changes in the integument of *Clinostomum* species following nanoparticle treatment using scanning electron microscopy (SEM), providing valuable insights into the mechanism of action of these novel antiparasitic agents. Additionally, this study conducted an in vitro analysis of the mortality rates of different nanoparticles against fish-borne zoonotic parasites, specifically *P. vivax* and clinostomid MCs.

## Materials and methods

### Ethical approval

The study protocol complied with the guidelines for the use of fish in research and was approved by the Institutional Animal Care and Use Committee (IACUC) of the Faculty of Veterinary Medicine, Cairo University (Vet CU. IACUC 03,162,023,644).

### Study area and fish samples

The current study was conducted from August 2023 to February 2024. A total of 453 Nile Tilapia (*O. niloticus*) were examined, collected randomly from the Nile River in Giza (29°59′13.2"N, 31°12′42.5"E) and Meet Damses (Dakahlia Governorate) (30°49′35.94"N, 31°13′21.40"E). The wild *O. niloticus* samples ranged in body weight from 100 to 199 g and were collected from water drains close to fish farms, where the fishermen had observed abnormal yellow grubs in the buccaneer cavity and on the fish’s skin. The Nile tilapia, including both wild and farmed individuals, were collected by fishermen and transferred alive in plastic bags with oxygen supply to the Parasitology Laboratory, Faculty of Veterinary Medicine, Cairo University, for further examination within the minimum time delay. Fish were maintained in the laboratory in aerated aquarium containing de chlorinated tap water.

### Parasitological examination

Fish were euthanized by overdosing with MS-222 anesthetic (Sigma), sacrificed, and carefully examined to detect parasitic infections following standard protocols^19^. Two types of cysts, microscopic and macroscopic, were investigated in the examined fish specimens using a hand lens, naked eye, and a stereo microscope, as described by Mahdy et al.^11^**.** The microscopic metacercariae (MC) were recovered, excised by breaking the cyst wall using a sharp needle, and washed in physiological saline-filled Petri dishes^22^**.** The macroscopic MCs were fixed between two glass slides in 70% ethanol. The parasites were stained with acetic acid alum carmine, dehydrated with graded concentrations of alcohol, cleared in clove oil, mounted in Canada balsam, and examined by light microscopy. The encysted metacercariae (EMCs) were identified based on international keys for the families Clinostomidae^23^, Euclinostomidae^24^, and Prohemistomidae^25^.

#### Scanning electron microscope

The MCs of *Clinostomum* and *Euclinostomum* species, collected from the buccaneer cavities and kidneys of the investigated fishes, respectively, were thoroughly washed with 0.9% NaCl and then fixed with 2.5% glutaraldehyde. The larvae were subjected to a dehydration series using increasing concentrations of ethanol (50% to 100%). The worms were completely dried using a CO2 critical point dryer (Autosamdri-815, Germany) and then cut at the anterior and posterior ends, mounted on stubs, and coated with 20 nm of gold. The worms were imaged using a scanning electron microscope (JSM 5200, electron probe) and a micro-analyzer (Jeol, Japan) at the Faculty of Agriculture, Cairo University^26–28^.

#### Structural characterization of nanoparticles

The chitosan, selenium, and silver nanoparticles were characterized using Transmission Electron Microscopy (TEM). Imaging was performed with a JEM-2100 TEM (JEOL, Japan) operating at 80 kV. The samples were sonicated in ethanol, deposited onto copper-coated carbon grids, and allowed to evaporate. The nanoparticles were purchased from nanotech Egypt® and characterized at the Faculty of Agriculture, Cairo University.

### Determination of LC50 and LC90 of nanoparticles against EMCs

Different concentrations (1–32 µg/ml) of the nanoparticles (chitosan, selenium, and silver) were tested against the EMCs of *Prohemistomum, Clinostomum,* and *Euclinostomum* species. Each concentration was applied in 8–10 replicates in tissue culture plates, with 10 freshly prepared EMCs in buffered phosphate saline (pH 7.2) per replicate. The EMCs were immersed in 100 ml (*Prohemistomum*) or 10 ml (*Clinostomum* and *Euclinostomum*) of each dilution for 10–20 min with continuous stirring. The treated and control EMCs were then removed, preserved in buffered phosphate saline (pH 7.2), and examined for live or dead individuals. Mahdy et al.^12^.

### Statistical analysis

Significant differences in the detected values between the control fish and other experimental groups were determined using one-way ANOVA, followed by an appropriate post-hoc test for pairwise comparisons. The SPSS software, Version 17.0 (https://www.ibm.com/products/spss-statistics) with significance level of P < 0.0.^29^. (SPSS Inc., IBM, Chicago, IL, USA) was used, with a significance level of P < 0.05.

## Results

### The cross-sectional analysis of different EMC collected in different governorates in Egypt

The collected fish were evaluated for the presence of EMCs during the period from 10 August 2023 to 9 February 2024 from investigated governorates were infected with EMC either with one species as; a single or mixed infection. The current study found mixed infection with more than one cyst-type infection in Nile tilapia; (Clinostomid and Euclinostomid) or (*Prohemistomum* and *Clinostomum* sp*.*). Furthermore, the most common microscopic type of EMC; are Prohemistomid EMCs in *O. niloticus* from Giza. During the inspected period, a total of 453 investigated random samples of fish for examination of fish-borne zoonotic trematodes. A total of prevalence rate of infection with EMC was (453/185 = 40. 8%) of *O*. *niloticus* from Giza and Dakahlia were collected and displayed in the Table 1, 2. Two different governorates were evaluated for the presence of microscopic and macroscopic EMCs; from Giza and Dakahlia. The prevalence rate of *O. niloticus* infection was 34.11% and 49.5% in Giza and Dakahlia, respectively. The most prominent postmortem findings were the presence of characteristic large yellow cysts in the investigated fish specimens embedded in the buccaneer cavities, kidneys, and body cavity attached to visceral organs. Muscles and other internal organs were free from parasitic cysts.

**Table 1:** Prevalence of FBZT-infected fish with zoonotic metacercarial cysts.

Governorate	FISH	No. of Ex fish	No. of inf. fish	%	Type of EMC
Giza	*O. niloticus*	255	87	34.11	Clinostomid Euclinostomid Prohemistomid
Dakahlia	*O. niloticus*	198	98	49.5	Clinostomid Euclinostomid
Total		453	185	40.8

**Table 2:** Prevalence types of zoonotic metacercarial cyst in investigated freshwater fish.

Type of EMC	Total Ex. No	No. of inf. fish	*Clinostomum* spp.		*Euclinostomum* spp.		*Prohemistoum* sp*.*	
			Inf	%	No. of inf. fish	%	No. of inf. fish	%
Giza	255	87	34	39.08	15	17.2	31	35.56
*Dakahlia*	198	98	43	43.87	21	21.41	22	22.44
*Total*	453	185	77	41.62	36	36.73	53	28.64

Active-free metacercariae were liberated after squeezing the detected EMCs. Other postmortem lesions were extensive hemorrhagic patches surrounding the metacercarial attachment sites and congestion of kidneys.

**Fig. 1:**
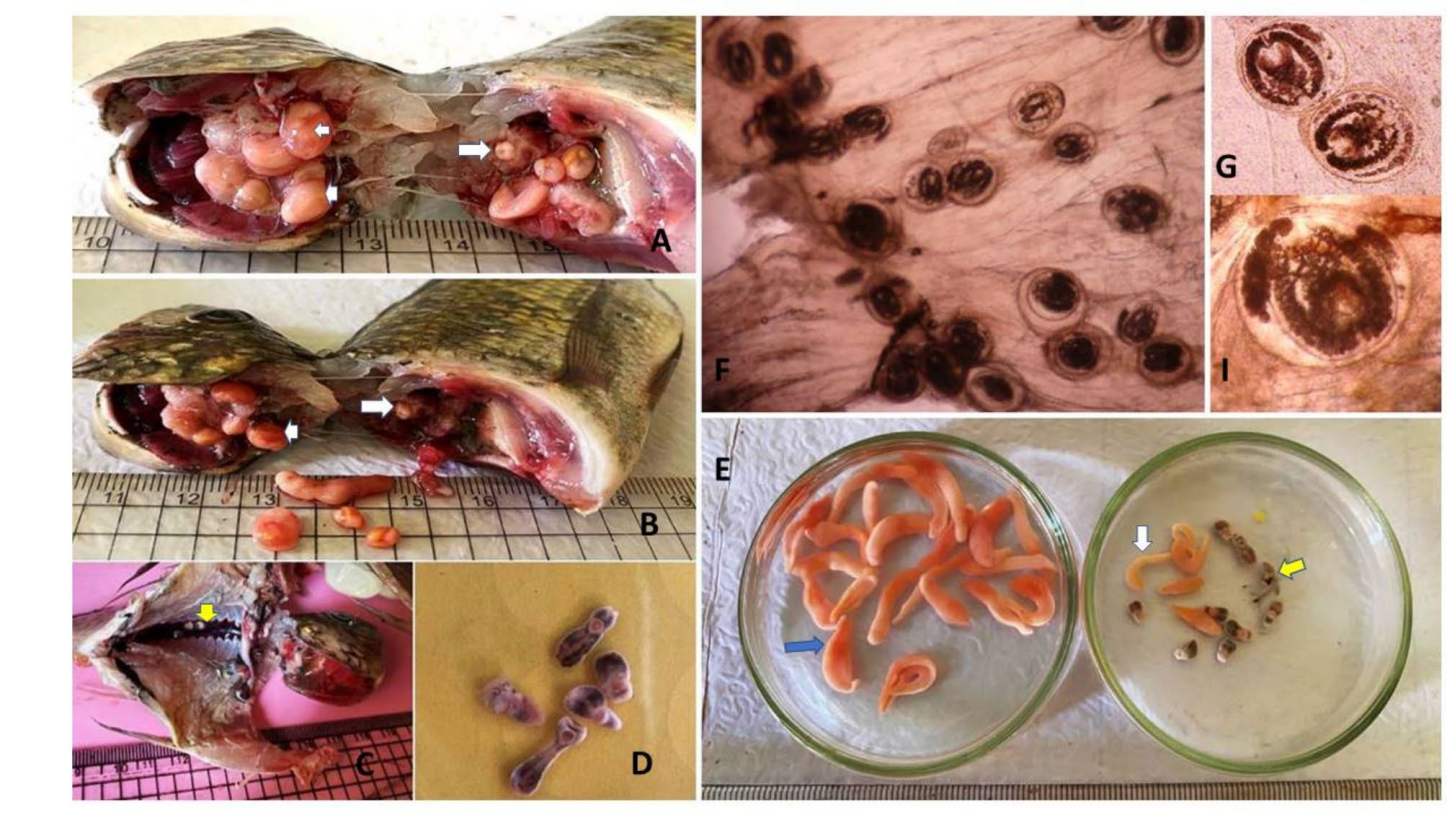
(**A**) Fresh *O. niloticus* under investigation that infected buccal cavities with clinostomide cysts; *C. phalacrocrasis* (head arrows) and *C. complanatum* (arrow) (**C**) Fresh *O. niloticus* under investigation that infected Kidneys with *Euclinostomum* cysts (arrow). (**D**) Excysted *Euclinostomum* metacercariae. (**E**) Excysted *C. phalacrocrasis* metacercariae (blue arrow), *C. complanatum* (white arrow), *Euclinostomum* metacercariae (yellow arrow).

Infection sites varied between Clinostomidae species. The macroscopic parasites were commonly noticed in the buccal cavity of tilapia, with lower infection levels in the abdominal cavity. The sites listed from highest to lowest were as the following: branchial cavity > buccaneer cavity > kidneys. The types of EMCs of *C. phalacrocoracis* and *C. complanatum* were noticed in the branchial cavity > buccal cavity. *E. heterostomum* was noticed in the Kidneys of investigated fishes (Fig. 1A-D). The freshly microscopic encysted *Prohemistomum* metacercarial stage was exhibited the relatively motile, with a high degree of contractility and flexibility inside cyst wall. In addition, the freshly macroscopic excysted Clinostomid; *Clinostomum* and *Euclinostomum* worms were relatively stout and motile, with a high degree of contractility and flexibility. The worm’s body was truncated anteriorly and broadly rounded posteriorly. Bodies were divided by a slight constriction into narrower pre-acetabular and broader posterior parts. The morphometric characteristics of all recovered metacercarial species from farmed and wild freshwater fishes. The mean intensity of metacercarial intensity were done.

### Identification of the obtained metacercariae (EMCs)

#### A- Microscopic EMCs; Prohemistomum vivax (*Cyathocotylidae*; Sonsino, 1892)

The obtained EMCs were found heavily distributed in skeletal muscles and organs of infected fishes (*O.* *niloticus*) with an intensity rate that ranged from 9–21 cysts (12 ± 0.9)/ microscopic field (× 4). These cysts were double-walled, spherical, having a thick outer shell and inner hyaline layer with a maximum diameter of 343- 449 μm. (Fig. 1F,G,I).

### B- Macroscopic EMCs

#### Prevalence and intensity of Clinostomid metacercariae (CMCs)

The prevalence and pattern of the intensity of Clinostomid MCs in investigated tilapia specimens (Fig. 1E). The prevalence of CMC was higher in farmed fish (35.00%) than in wild tilapia (24.05%). The intensity of parasites varied between investigated tilapia specimens. The mean parasite intensity per host was higher (7.85) in farmed fish than in wild tilapia (7.2). The highest number of MCs collected from a single fish was 19 cysts. Types of FBZT infected fish with CMCs were revealed**.** The intensity rate of *Clinostomum* sp. and *Euclinostomum* MCs was displayed in Table 3.

**Table 3:** Prevalence, mean intensity of clinostomid MC in farmed and wild Nile tilapia from Dakahlia governorate.

Fish species	(1)	(2)	%	No. Ps	3	Pattern of parasites intensity		Fish organs infected with clinostomidmetacercarial species			
						I	%	Infected organ	*C. phal*	*C. com*	*E. het*
Wild tilapia	158	38	24.0	163	7.2	*	12 (7.4%)	Skin	-	-	-
								Buccal cavity	√	√	-
						**	15 (9.2%)	Buccal cavity	√	√	√
						***	10 (6.13%)	Kidneys	-	-	√
						****	1 (0.6%)	Abdominal cavity	-	√	√
Farmed tilapia	140	49	35.0	181	7.85	*	2 (1.1%)	Skin	-	√	√
								Branchial cavity	√	√	-
						**	9 (4.97%)	Buccal cavity	√	√	√
						***	10 (5.5%)	Kidneys	-	-	√
						****	4 (2.20%)	Abdominal cavity	-	√	√

No. exam fish (1),No. infected fish (2),No. parasites, Mean intensity = 3. I (Intensity), *Minimum (1–2 cyst), ** medium (3–5 cyst), *** High (6-10cyst), **** Max (> 10 cyst), Mean for intensity of infection*.phalacrocoracis*)*, C. complanatum, E. heterostomum.*

### Characterization of the produced Nano-particles

#### Chitosan Nano-particles

**Fig. 2:**
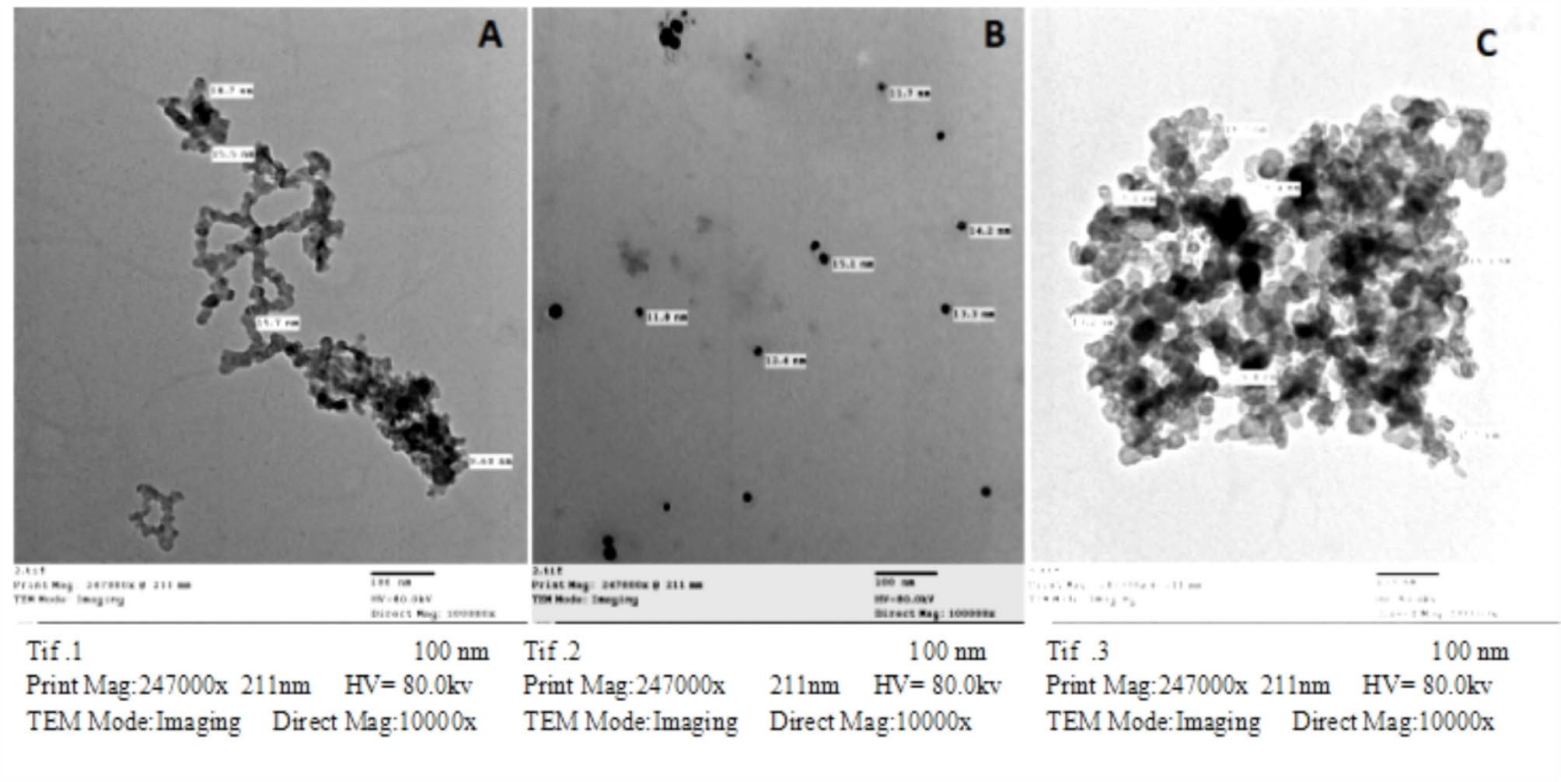
Transmission electron microscopic characterization of Chitosan, silver and Selenium nanoparticles showing its (**A**) Chitosan NPs; rounded in shape and its size which ranged from 9.6–18.7 nm. (**B**) Silver NPs; rounded in shape and its size which ranged from 11.7–15.1 nm (**C**) Selenium NPs; rounded in shape and its size which ranged from 13.2- 19.8 nm.

The morphology of the chitosan was investigated by TEM. The chitosan nanoparticles were observed to be nearly spherical with an average diameter of 9.6–18.7 nm (Fig. 2A).

#### Silver Nano-particles

The morphology of the silver nanoparticle was investigated by TEM. The silver nanoparticles were nearly spherical with an average diameter of 11.7–15.1 nm (Fig. 2B).

#### Selenium nanoparticles

The morphology of the Selenium nanoparticle was investigated by TEM. The Selenium nanoparticles were clearly observed to be nearly spherical with an average diameter of 13.2- 19.8 nm (Fig. 2C).

### In vitro anti parasitic activity of three types of nanoparticles on Clinostomid MCs

**Fig. 3:**
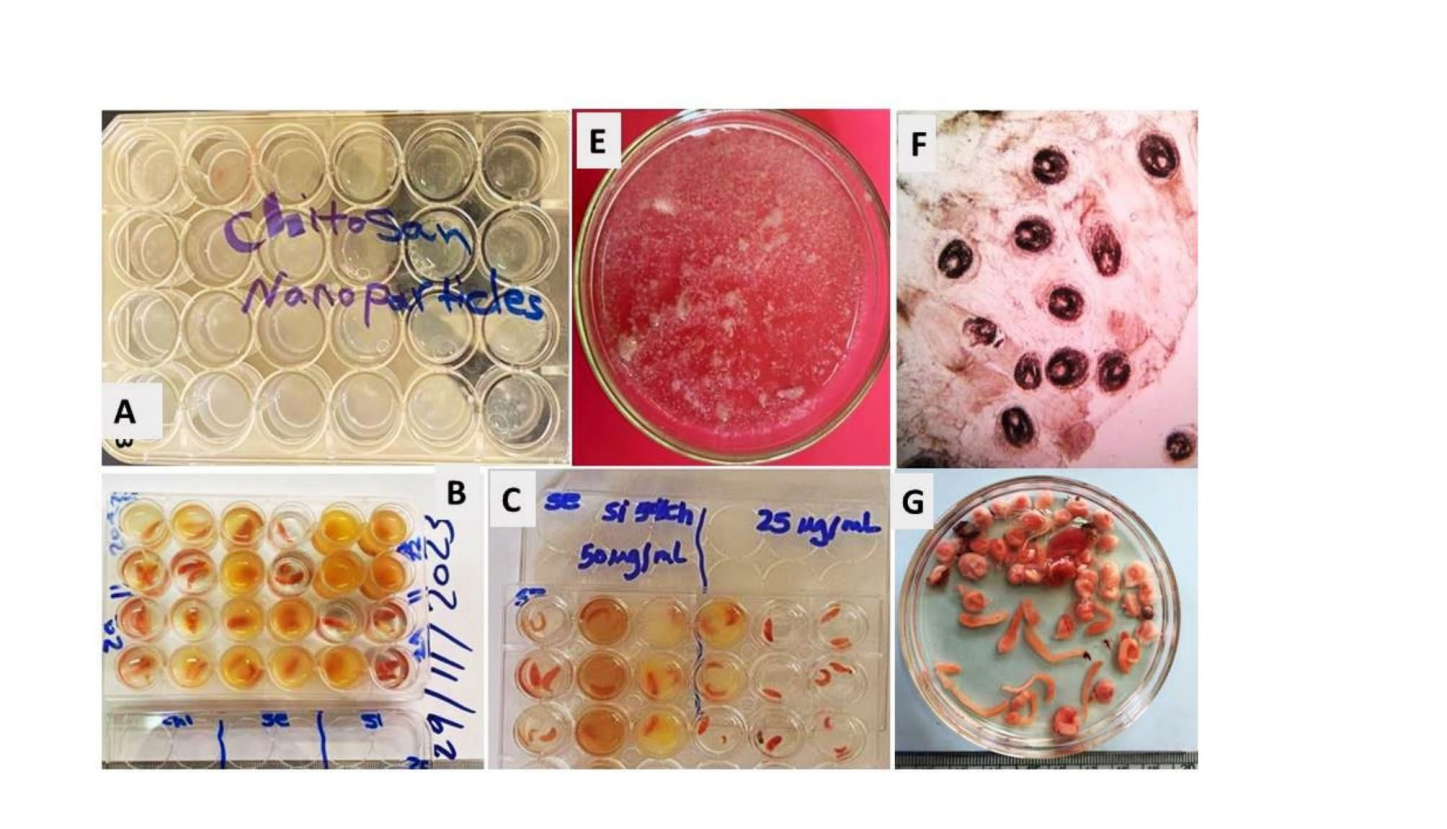
(**A-C**) Tissue culture plate used for determination of different concentrations (12.5, 25, 50 & 100 mg/ml) of different three NPs; Silver (Si), Selenium (se), and Chitosan( ch). (**E**) Isolated microscopic cysts of *Prohemistomum* sp. from infected *O. niloticus* in Petri dish. (**F**) Microscopic *Prohemistomum* cysts microscopically field under assessment (× 10). (**G**) Clinostomid cysts and freshly excysted metacercariae macroscopically under assessment.

All data of three types of nanoparticles the vitro study on *Clinostomum* spp. MCs using are displayed in Tables 4, 5, 6, 7 and Fig. 3.

**Table 4:** Mortality bioassay using Chitosan nanoparticles on *Clinostomum* spp.

Tested concentration	Mortality bioassay using Chitosan nanoparticles				
	M.M. % ± S. D				
	10 min	20 min	30 min	1 h	2 h
100 μg/ml	77.7 ± 0.12	83.3 ± 0.04	100 ± 0.67	100 ± 1.07	100 ± 0.85
50 μg/ml	62 ± 1.6	72 ± 1.06	88.8 ± 1.98	100 ± 2.13	100 ± 2.91
25 μg/ml	45 ± 1.03	61 ± 1.08	83 ± 2.11	100 ± 1.04	100 ± 1.00
12.5 μg/ml	16 ± .09	28 ± 1.53	66 ± 1.05	83 ± 1.06	100 ± 0.07
Negative Control	0 ± 0.00	0 ± 0.00	0 ± 0.00	0 ± 0.00	0 ± 0.00

**Table 5:** Mortality bioassay using silver nanoparticles on *Clinostomum* MCs.

Tested concentration	Mortality using silver nanoparticles				
	M.M. % ± S. D				
	10 min	20 min	30 min	1 h	2 h
100 μg/ml	45 ± 1.09	75 ± 0.36	83 ± 0.17	100 ± 0.06	100 ± 1.01
50 μg/ml	16.6 ± 0.45	44 ± 0.92	61 ± 0.19	72.2 ± 0.13	100 ± 2.01
25 μg/ml	0 ± 2.04	16.6 ± 0.11	44.5 ± 0.27	55.5 ± 0.15	72.2 ± 1.06
12.5 μg/ml	0 ± 0.00	0 ± 0.00	16 ± 0.90	27 ± 0.9	44 ± 3.01
Negative Control	0 ± 0.00	0 ± 0.00	0 ± 0.00	0 ± 0.00	0 ± 0.00

**Table 6:** Mortality bioassay using Selenium nanoparticles on *Clinostomum* MCs.

Tested concentration	Mortality bioassay using Selenium nanoparticles				
	M.M. % ± S.D				
	10 min	20 min	30 min	1 h	2 h
100 μg/ml	44 ± 0.02	60 ± 2.01	72 ± 0.87	83 ± 0.21	93 ± 0.21
50 μg/ml	28 ± 0.03	38 ± 2.03	38 ± 0.12	45 ± 1.02	61 ± 1.02
25 μg/ml	16 ± 0.08	27 ± 1.02	44.5 ± 0.08	45 ± 0.12	45 ± 0.98
12.5 μg/s%	0 ± 0.00	16 ± 0.23	27 ± 0.11	27 ± 0.87	44 ± 0.90
Negative Control	0 ± 0.00	0 ± 0.00	0 ± 0.00	0 ± 0.00	0 ± 0.00

**Table 7:** Comparative analysis of mortality bioassay using Chitosan, Silver, and Selenium nanoparticles on Clinostomum MCs.

Nanoparticle	LC50 (Conc./time)	LC90 (Conc./time)
1. Chitosan	12.5 μg/ml/30 min	12.5 μg/ml/2 h
2. Silver	25 μg/ml/1 h	50 μg/ml/2 h
3. Selenium	50 μg/ml/2 h	100 μg/ml/2 h

#### Chitosan NPs Mortality bioassay study (LC50)

**Fig. 4:**
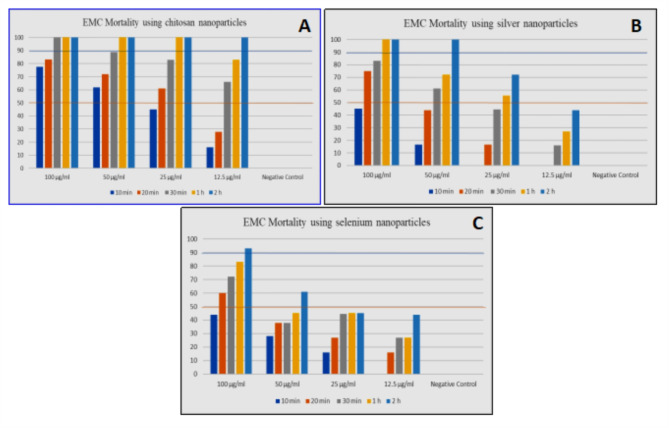
(**A**) Mortality bioassay using Chitosan nanoparticles on *Clinostomum* MCs (**B**): Mortality bioassay using silver nanoparticles on *Clinostomum* MCs. (**C**): Mortality bioassay using Selenium nanoparticles on *Clinostomum* MCs.

The vitro study on *Clinostomum* spp. MCs using Chitosan reveal that; the mortality were recorded in 12.5 μg/ml at 30 min. The mortality increased with increasing concentration and times; the LC50 was recorded at 66 μg/ml for 30 min exposure times and the LC90 was recorded at 100 for 120 min at a concentration of 12.5 μg/ml. LC90 was recorded in100 ppm after 2 h’s exposure time Fig. 4A

#### Silver NPs Mortality bioassay study (LC50)

The vitro study on *Clinostomum* MC using silver NPs reveal that; the mortalities were recorded in 25 μg/ml at 60 min. The mortality increased with increasing concentration and times; the LC50 was recorded 55.5% after 60 min exposure times and the LC90 was recorded at 100 for 120 min at a concentration of 50 μg/ml. LC90 was recorded in100 ppm after 2 h’s exposure time Fig. 4B.

#### Selenium NPs Mortality bioassay study

The vitro study on *Clinostomum* MCs using *Selenium nanoparticles* reveal that; the mortality were recorded in 50 μg/ml at 2 h. The mortality increased with increasing concentration and times; the LC50 was recorded at 61.0% for 2 h exposure times and the LC90 was recorded at 100 for 2 h at a concentration of 100 μg/ml. LC90 was recorded in100 ppm after 2 h exposure time Fig. 4C.

#### Mortality bio-assay study of chitosan NPs on Prohemistomum vivax EMCs

The in vitro study on *P. vivax* EMCs using chitosan NPs reveals that; no moralities were recorded in 1 μg/ml; while in 2 μg/ml the mortality was recorded after half hour exposure period (10.0 μg/ml ± 0.00); with increasing the concentration; the mortality increases as in 4 μg/ml the mortality begins for 20 min and increasing with the time increase. The mortality increased at the concentration of 8 μg/ml for 10 min with 10.0% mortality and increased reached 2 h. In concentrations of 8 μg/ml for 1 h the mortality rate occurred (LC50), (Table 8). In addition, in concentrations 16 μg/ml, the mortality increased to LC90 for a 2 h exposure period.

**Table 8:** Mortality bioassay study of chitosan NPs on *EMCs* (*P. vivax*).

Tested concentration	*EMC* Mortality using Chitosan NPs				
	M.M. % ± S.D				
	10 min	20 min	30 min	1 h	2 h
32 μg/ml	30.0 ± 0.79	60 ± 0.19	100 ± 0.00	100 ± 0.00	100 ± 0.00
16 μg/ml	16 ± 0.46	40.00 ± 0.34	50.00 ± 0.0	73.0 ± 0.50	100 ± 0.00
8 μg/ml	10.0 ± 0.36	20.0 ± 0.42	37.0 ± 0.25	52 ± 0.75	60.0 ± 0.50
4 μg/ml	00 ± 0.00	15 ± 0.00	26.0 ± 0.69	35.0 ± 0.50	40.0 ± 0.57
2 μg/ml	00 ± 0.00	00 ± 0.00	10.0 ± 0.00	15.0 ± 0.00	30 ± 0.00
1 μg/ml	00 ± 0.00	00 ± 0.00	00 ± 0.00	00 ± 0.00	00 ± 0.00
Negative Control group with no treatment	00 ± 0.00	00 ± 0.00	00 ± 0.00	00 ± 0.00	00 ± 0.00

*M.M % = mean mortality percentage ± standard deviation.

#### Mortality bio assay study of Silver NPs

The in vitro study on *P. vivax* EMCs using *silver NPs* reveals that; no moralities were recorded in 1 & 2 μg/ml; while in 4 μg/ml the mortality was recorded after 10 min exposure period (5.0 μg/ml ± 0.36); with increasing the concentration; the mortality increases as in 8 μg/ml the mortality begins for 10 min and increasing with the time increase. The mortality increased at the concentration of 16 μg/ml for 1 h with 55.0.50% mortality and increased reached 2 h. In concentrations of 16 μg/ml for 1 h, the mortality rate occurred (LC50). In addition, in concentrations of 32 μg/ml, the mortality increased to LC90 for a 30 min exposure period (Table 9).

**Table 9:** Mortality bio assay study of *silver NPs* on *EMCs* (*P. vivax*).

Tested concentration	*EMC* Mortality using silver NPs				
	M.M. % ± S.D				
	10 min	20 min	30 min	1 h	2 h
32 μg/ml	35 ± 0.79	70 ± 0.19	100 ± 0.00	100 ± 0.00	100 ± 0.00
16 μg/ml	10 ± 0.46	40.00 ± 0.34	42.00 ± 0.0	55 ± 0.50	100 ± 0.00
8 μg/ml	5.0 ± 0.36	20 ± 0.42	30.0 ± 0.25	40 ± 0.75	45 ± 0.50
4 μg/ml	00 ± 0.00	00 ± 0.00	20.0 ± 0.69	26.0 ± 0.50	33.0 ± 0.57
2 μg/ml	00 ± 0.00	00 ± 0.00	00 ± 0.00	00 ± 0.00	00 ± 0.00
1 μg/ml	00 ± 0.00	00 ± 0.00	00 ± 0.00	00 ± 0.00	00 ± 0.00
Negative Control group with no treatment	00 ± 0.00	00 ± 0.00	00 ± 0.00	00 ± 0.00	00 ± 0.00

*M.M % = mean mortality percentage ± standard deviation.

#### Mortality bio assay study of Selenium NPs

The in vitro study on *P. vivax* EMCs using selenium NPs reveals that; no mortalities were recorded in 1 & 2 μg/ml; while in 4 μg/ml the mortality was recorded after 10 min exposure period (5.0 μg/ml ± 0.36); with increasing the concentration; the mortality increases as in 8 μg/ml the mortality begins for 10 min and increasing with the time increase; (Table 10).

**Table 10:** Mortality bio assay study of *selenium NPs* on *EMCs* (*P. vivax*).

Tested concentration	EMC Mortality using selenium NPs				
	M.M. % ± S.D				
	10 min	20 min	30 min	1 h	2 h
32 μg/ml	30 ± 0.89	70 ± 0.15	00	00	00
16 μg/ml	25 ± 0.55	50.0 ± 0.56	25.0 ± 0.55	00	00
8 μg/ml	15 ± 0.54	32 ± 0.45	40.0 ± 2.45	13 ± 0.34	00
4 μg/ml	5.0 ± 0.36	15 ± 0.35	35.0 ± 3.69	45 ± 0.55	00
2 μg/ml	00 ± 0.00	00 ± 0.00	00 ± 0.00	00 ± 0.00	00 ± 0.00
1 μg/ml	00 ± 0.00	00 ± 0.00	00 ± 0.00	00 ± 0.00	00 ± 0.00
Negative Control group with no treatment	00 ± 0.00	00 ± 0.00	00 ± 0.00	00 ± 0.00	00 ± 0.00

*M.M % = mean mortality percentage ± standard deviation.

#### SEM Results

**Fig. 5:**
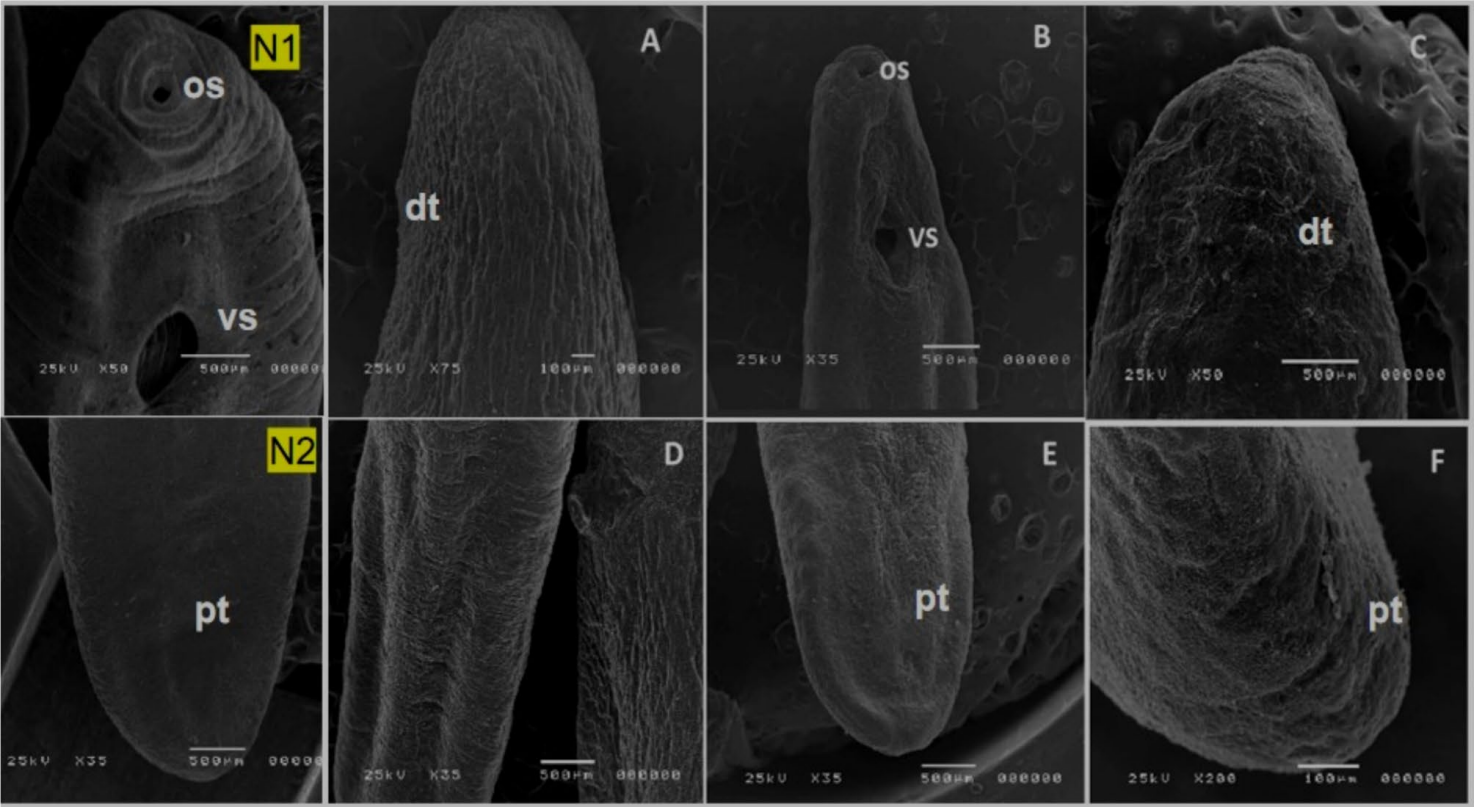
SEM micrograph of *Clinostomum* spp. N1) Normal *Clinostomum* larva, anterior tegumental surface (ventral view) exhibited; visible smooth transverse annulations, oral sucker (os) with collar-like rings and ventral sucker (vs). N2) dorsal view of posterior tegument (dt) hind body, dorsal view exhibited the smooth posterior tegument (Pt). (**A-D**) Treated C*linostomum* with chitosan NPs, the body is severing longitudinal damage after treated at dose of (LC90%). (**B,E**) SEM micrograph of treated dorsal and ventral views of *Clinostomum* spp. with Silver NPs showed shrinkage and dislocated of suckers; oral sucker (os), ventral sucker (vs). (**C,F**) SEM micrograph of treated dorsal and ventral views of *Clinostomum* spp. with Seleinum NPs showed damage and shrinkage in anterior and posterior tegumental surfaces.

The worms were exposed to various concentrations of nanoparticles; in contrast to the control worms, which were viable and moving with great activity, chitosan, silver, and selenium exhibited degenerative effects that increased with dose and exposure time. When MC was exposed to Chitosan NPs, the mortality rate was considerably higher than when worms were exposed to Silver and Selenium NPs. (Fig. 5); display the ultra-structure observation of worms from the control, unexposed group. There were additionally visible smooth transverse annulations and ridges on control worms (unexposed to NPs). The oral sucker was made up of two separate, collar-like rings that were flat and semicircular in shape and had sensory papillae attached to them. A sizable closed ventral sucker was present on the ventral surface, close to the oral sucker. A distinct ventral fold with a sponge-like character surrounded the ventral sucker, and dome-like papillae were present around the fold margins (Fig. 5N1). The worms had a distinct, marked smooth and normal tegument structure at the hind body (Fig. 5N2).

The ultra-structure observation of worms were exposed to three types of NPs; Chitosan, Silver & Selenium at dose 12.5 μg/ml at 30 min, 25 μg/ml/ h & 50 μg/ml at 2 h. (LC50) generally un figuration of integument surface all over the worms are shown in Fig. (5). The worms from Chitosan NPs exposed group at LC50, mortality rate occurred are shown in Figs. (5A,D). Ultra—structure description of marked disappearance of the transverse ridges’ striations and shrinkage of integument surface in dorsal surface of fore and hind bodies. The dislocation of the two suckers of the parasite. The worms exposed to Silver NPs at LC50, the mortality rate occurred are shown in Fig. (5B,E) of marked disappearance of the transverse ridges’ striations and dislocation of the two suckers of the parasite. The worms from 3^rd^ exposed gp that exposed to Selenium NPs, exhibited swollen and numerous blebs on the integument surface as compared to the control specimen’s fore body and hind body (Fig. 5C, F).

## Discussion

The substantial economic losses and significant public health concerns associated with food-borne zoonotic trematode parasites have led to a growing apprehension regarding these pathogens in developing nations^11,22^. These parasitic infections can severely impair the growth, health, physiology, and productivity of fish populations^30,31^. Furthermore, geographical variations, the presence of diverse cercariae varieties, and the migratory patterns of birds can influence the intensity of infection, which is also contingent upon the degree of pollution from bird excrement containing the fluke’s eggs^7,32^. Other studies have shown that the development of the host’s defense mechanisms against parasites is aided by the stimulation of humoral host immune responses^5^.

Nanotechnology has emerged as a transformative force across various industries, including biomedicine, where it is utilized for applications such as antimicrobial drugs, catalysts, electronics components, and optical fibers^21^. Nanoparticles are employed for drug and gene delivery and play a significant role in tissue engineering and parasite therapy. Nanomaterials are increasingly being used in cost-effective, environmentally friendly products^33^. Nanoparticles have recently been employed as antiparasitic agents to treat a variety of parasites. The World Health Organization (WHO) has released evidence-based guidelines for controlling vectors and safeguarding humans against infection^4^. Silver nanoparticles (NPs) are particularly promising due to their antibacterial properties and their efficacy against various microbial disease agents^34^. Among the various mechanisms by which metal nanoparticles combat bacteria, one potent method involves adhering to and disrupting the cell wall and membrane through electrostatic interactions. They may also obstruct ion transit by interacting with ions and ion channels^35^. The use of nanoparticles as drug carriers presents a promising approach for treating a wide variety of diseases^36,37^.

Chitosan, a polysaccharide naturally present in the exoskeleton of insects and crustaceans, consists of randomly dispersed β-linked D-glucosamine and N-acetyl-D-glucosamine^38^. It exhibits desirable qualities such as biocompatibility, biodegradability, non-toxicity, and bioadhesiveness^38^. These properties have led to the widespread use of chitosan-based formulations, primarily in the form of emulsions, for various applications, including medicine administration, edible coatings for aquaculture species, and human medical uses such as dentistry and surgery^39^. Colloidal silver nanoparticles are one of the primary nanotechnology products employed to combat a diverse array of diseases, including bacterial, viral, parasitic, and fungal infections, owing to their wide-ranging effects, ease of application, crystallographic structure, high surface-to-volume ratio, and compatibility with multiple compounds^40^. Various metal nanoparticles, including copper, titanium, and silver, have been employed in the prevention and treatment of diseases.

The use of chitosan-based systems for encapsulation and delivery in aquaculture has been well-documented. In this study, the authors investigated the larvicidal effects of chitosan, silver, and selenium nanoparticles against Clinostomum larvae, a parasitic trematode that infects various fish species. The calculated LC50 values revealed that chitosan nanoparticles were the most effective, with an LC50 of 12.5 μg/ml resulting in 66% mortality after 30 min and an LC90 at the same concentration after 2 h of exposure. These findings align with previous studies that have reported the anthelmintic properties of chitosan. For instance, Mostafa et al.^41^ found chitosan to be an effective anthelmintic, while Abdel-Latif et al.^42^ and Salem et al.^43^ observed that chitosan particles decreased the number of adult and egg parasites, such as *Hymenolepis nana* and *Ascaridia columbae*, and caused deformation in the worms’ body cuticles and cephalic region.

In contrast, the LC50 values for silver and selenium nanoparticles were higher, at 25 μg/ml after 1 h and 50 μg/ml after 2 h, respectively. These results differ from the findings of Abdelsalam et al.^44^, who reported higher toxicity of nanosilver structures derived from crab shells against *Anopheles stephensi*, with LC50 values ranging from 3.18 to 6.54 ppm. Scanning electron microscopy (SEM) analysis revealed the specific effects of the different nanoparticles on *Clinostomum* larvae. Chitosan nanoparticles caused longitudinal damage to the dorsal integument surface, silver nanoparticles led to dislocation of suckers, and selenium nanoparticles resulted in damage and shrinkage to the anterior and posterior tegumental surfaces. The small size of the nanoparticles (9.6–18.7 nm) may have allowed them to penetrate the cuticle of the worms, increasing permeability and leading to their mortality.

The antiparasitic and antibacterial effects of chitosan-silver nanocomposites have been further confirmed in other studies, such as the treatment of *Pseudolynchia canariensis* infestations in pigeons^45^. Additionally, plant extracts like *Verbena alternifolia* and *Mentha piperita* have also shown strong antiparasitic effects on the tegumental surface of *Euclinostomum* and *Clinostomum* worms^24,27,28^. In a separate in vitro study, the authors found that chitosan nanoparticles were effective against *P. vivax* cysts, with an LC50 of 8 μg/ml after 1 h and an LC90 at 16 μg/ml after 2 h of exposure. These results are in line with the findings of Abou Shady et al.^26^ and Abdel-Latif et al.^42^, who reported that chitosan particles and Carica papaya seeds decreased the numbers of adult and egg *Hymenolepis nana* infecting mice. This study highlights the superior larvicidal efficacy of chitosan nanoparticles against *Clinostomum* larvae, outperforming silver and selenium nanoparticles. The nanoparticles’ small size and ability to penetrate the worms’ cuticle appear to be key factors in their potent antiparasitic effects. These findings contribute to the growing body of evidence supporting the use of chitosan-based nanomaterials for parasite control in aquaculture and other applications.

## Conclusion

This study investigates the use of nanotechnology to control infections by the flatworm parasite *Clinostomum* in freshwater fish. In Egypt, these parasites, known as "yellow grub disease," have a negative impact on commercial fisheries. *Clinostomum* and related parasites have complex life cycles, encysting in fish muscle and tissues as infective stages. The researchers evaluated three different nanoparticles—chitosan, silver, and selenium—for their antiparasitic effects on *Clinostomum* larvae in vitro. Scanning electron microscopy (SEM) was used to assess the effects of the nanoparticles on the parasites’ surface tegument (outer layer). The results showed that chitosan nanoparticles were more effective than silver or biodegradable selenium nanoparticles in controlling *Clinostomum* and related *Prohemistomum* larvae. Chitosan nanoparticles at a concentration of 12.5 μg/ml demonstrated a promising antiparasitic effect. Further in vivo studies are recommended to develop effective and safe anthelmintic (anti-parasitic) treatments using chitosan nanoparticles to overcome such parasitic infections in fish. The novel use of nanotechnology provides a potential new tool for managing important parasitic diseases in aquaculture.

## Data Availability

All the authors declare that; all the data supporting the results rep orted in our article were found included in this article only.

## References

[1] Shamsi, S. Seafood-borne parasitic diseases: a “one-health” approach is needed. *Fishes* **4**(1), 9. 10.3390/fishes4010009 (2019).

[2] Shamsi, S. et al. Wild fish as reservoirs of parasites on Australian Murray cod farms. *Aquaculture*. 539. ISSN 0044–8486. 10.1016/j.aquaculture.2021.736584 (2021)

[3] WHO. Workshop on food-borne trematode infections in Asia. Ha Noi, Vietnam. Hanoi, Vietnam, 26–28 WPRO, 58. (2004).

[4] WHO. World Health Organization Pre-harvest food safety. Report of a WHO consultation with the participation of the Food and Agriculture Organization of the United Nations and the Office International des Epizooties. Berlin, Germany (2017).

[5] Kandil, O. M. et al. Cystic echinococcosis: development of an intermediate host rabbit model for using in vaccination studies. *Exp. Parasitol.*10.1016/j.exppara.2019.107800 (2020). 31726054 10.1016/j.exppara.2019.107800

[6] Mahdy, O. A., Abdel-Maogood, S. Z., Abdelsalam, M. & Salem, M. A. A multidisciplinary study on *Clinostomum* infections in Nile tilapia: micromorphology, oxidative stress, immunology, and histopathology. *BMC Vet. Res.* **20**, 60. 10.1186/s12917-024-03901-7 (2024). 38378547 10.1186/s12917-024-03901-7 PMC10877748

[7] Mahdy, O. A., Salem, M. A., Abdelsalam, M., Shaheed, I. B. & Attia, M. M. Immunological and molecular evaluation of zoonotic metacercarial infection in freshwater fish: a cross-sectional analysis. *Res. Veterinary Sci.* **172**, 105239. 10.1016/j.rvsc.2024.105239 (2024). 10.1016/j.rvsc.2024.105239 38583195

[8] Mahdy, O. A., Ramadan, R. M. & Salem, M. A. Innovative molecular and immunological approaches of heterophyiasis infecting some Egyptian marketed fishes. *BMC Vet. Res.* **20**, 385. 10.1186/s12917-024-04226-1 (2024). 39215340 10.1186/s12917-024-04226-1 PMC11363687

[9] Kotb, H. L., Mahdy, O. A. & Shaheed, I. B. Parasitological and histopathological study of digenetic trematodes in mullets from Lake Qarun. *Egypt. Glob. Vet.* **13**(2), 202–208 (2014).

[10] Mahdy, O. A., Mahmoud, A. M. & Abdelsalam, M. Morphological characterization and histopathological alterations of homologs Heterophyid metacercarial coinfection in farmed mullets and experimental infected pigeons. *Aquacult. Int.* **28**, 2491–2504. 10.1007/s10499-020-00602-4 (2020).

[11] Mahdy, O. A. et al. Epidemiological study of fish-borne zoonotic trematodes infecting Nile tilapia with first molecular characterization of two heterophyid flukes. *Aquacult. Res.* **00**, 1–14 (2021).

[12] Mahdy, O. A., Abdelsalam, M. & Salem, M. A. Molecular characterization and immunological approaches associated with yellow grub trematode (Clinostomid) infecting Nile Tilapia. *Aquac. Res.*10.1155/2023/5579508 (2023).

[13] Nasser, M. The occurrence of *Prohemistomum vivax* infections in man with redescription of the parasite. *Lab. Med. Program* **2**, 135–149 (1941).

[14] Anh, K. T. et al. Prevalence, species distribution, and related factors of fish-borne trematode infection in Ninh Binh Province, Hindawi. *BioMed Res. Int.*10.1155/2019/8581379 (2019). 10.1155/2019/8581379 PMC6699318 31467915

[15] Chai, J. Y. *Human intestinal Flukes. From discovery to treatment and control* (Springer Nature B.V, 2019).

[16] Ibrahim, T. B. & Mahdy, O. A. In vitro and in vivo effects of Carica papaya seed extract on the ultrastructure of the tegument of *Prohemistomum vivax* (Sonsino, 1892) (Trematoda: Prohemistomatidae). *Int. J. Zool. Res.* **13**, 45–53 (2017).

[17] Abdelkhalek, S. et al. Molecular identification and histopathological alterations associated with Prohemistomum vivax encysted metacercariae infection in farmed African Catfish (Clarias gariepinus). *Egypt. J. Veterinary Sci.* **55**(4), 1055–1065 (2024).

[18] Abdelkhalek, S. et al. Alterations in histopathology and stress-associated gene expression induced by infection with Prohemistomum vivax encysted metacercariae in Nile tilapia. *Aquacult. Int.* **32**, 5107–5124. 10.1007/s10499-024-01418-2 (2024).

[19] Fernandes, M. et al. Polysaccharides and metal nanoparticles for functional textiles: a review. *Nanomaterials*10.3390/nano12061006 (2022). 35335819 10.3390/nano12061006 PMC8950406

[20] El Naschie, M. S. Nanotechnology for the developing world. *Chaos Solitons Fractals.* **30**, 769–773. 10.1016/j.chaos.2006.04.037 (2006).

[21] Malik, S., Muhammad, K. & Waheed, Y. Nanotechnology; A revolution in modern industry. *Molecules.* **28**(2), 661. 10.3390/molecules28020661 (2023). 36677717 10.3390/molecules28020661 PMC9865684

[22] Mahdy, O. A. & Shaheed, I. B. Histopathological study on the effect of *Renicola heroni* on the kidneys of giant heron Ardea goliath. *Helminthologia* **38**, 81–83 (2001).

[23] Salem, M. A., Abdel-Maogood, S. Z., Abdelsalam, M. & Mahdy, O. A. Comparative morpho-molecular identification of *Clinostomum phalacrocoracis* and *Clinostomum complanatum* metacercaria coinfecting Nile tilapia in Egypt. *Egypt. J. Aquat. Bio. Fish.* **25**(1), 461–476 (2021).

[24] Mahdy, O. A., Abdel-Maogood, S. Z., Mohammed, F. F. & Salem, M. A. Effect of *Verbesina alternifolia* and *Mentha piperita* oil extracts on newly excysted metacercaria of *Euclinostomum heterostomum* (Rudolphi, 1809) (Digenea: Clinostomatidae) from naturally infected kidneys of *Tilapia zillii* in Egypt. *J. Egypt. Soc. Parasitol. (JESP)* **47**(3), 513–521. 10.12816/Jesp.2017.77706 (2017).

[25] Saad, M. S., Amani, M. S., & Olfat, A. M. Prevalence of metacercarial infection in some marketed fish in Giza Governorate, Egypt. *J. Egypt. Soc. Parasitol.* **49**(1), 129–134 (2019).

[26] Abou Shady, O. M., Basyoni, M. M. A. & Mahdy, O. A. The effect of praziquantel and *Carica papaya* seeds on *Hymenolepis nana* infection in mice using scanning electron microscope. *Parasitol. Res.* **113**, 2827–2836. 10.1007/s00436-014-3943-4 (2014). 24849866 10.1007/s00436-014-3943-4

[27] Mahdy, O. A., Abdel-Maogood, S. Z., Abdelrahman, H. A., Fathy, F. M. & Salem, M. A. Assessment of *Verbesina alternifolia* and *Mentha piperita* oil extracts on *Clinostomum phalacrocoracis* metacercariae from *Tilapia zillii*. *Beni-Suef Univ. J. Basic Appl. Sci.* **11**(1), 48 (2022).

[28] Mahdy, O. A., Abdelsalam, M., Abdel-Maogood, S. Z., Shaalan, M. & Salem, M. A. First genetic confirmation of Clinostomidae metacercariae infection in *Oreochromis niloticus* in Egypt. *Aquac. Res.* **53**(1), 199–207 (2022).

[29] Salem, M. A., Olfat, A., Mahdy, M. S. & Reem, M. R. The phylogenetic position and analysis of *Renicola* and *Apharyngostrigea* species isolated from cattle egret (*Bubulcus ibis*). *Sci. Rep.*. 10.1038/s41598-023-43479-y (2023). 10.1038/s41598-023-43479-y PMC10533816 37759085

[30] Shareef, P. A. & Abidi Syed, M. A. Studies on the epidemiology and histopathology of Euclinostomum heterostomum (Trematoda; Digenea) infection in Channa punctata from North India. *J. Poli. Fisheries*. **23**, 133-140, https://api.semanticscholar.org/CorpusID:30004681 (2015).

[31] Ibrahim, M. M. et al. Dasyrhynchus giganteus plerocercoids encysting in the musculature of Indian halibut (Psettodes erumei):seasonal prevalence, morpho-molecular characterization, and histopathological alterations. *BMC Vet.Res.* **20**(1), 332 (2024). 10.1186/s12917-024-04156-y PMC11265158 39039589

[32] Mahmoud, A. M. et al. Infectious bacterial pathogens, parasites and pathological correlations of sewage pollution as an important threat to farmed fishes in Egypt. *Environ. Pollut.* **219**, 939–948. 10.1016/j.envpol.2016.09.044 (2016). 27720545 10.1016/j.envpol.2016.09.044

[33] Shang, J. et al. Recent developments in nanomaterials for upgrading treatment of orthopedics diseases. *Front. Bioeng. Biotechnol.*10.3389/fbioe.2023.1221365 (2023). 37621999 10.3389/fbioe.2023.1221365 PMC10446844

[34] Yılmaz, G. E. et al. Antimicrobial nanomaterials: a review. *Hygiene.* **3**(3), 269–290. 10.3390/hygiene3030020 (2023).

[35] Wang, L., Hu, C. & Shao, L. The antimicrobial activity of nanoparticles: present situation and prospects for the future. *Int. J. Nanomedicine.* **12**, 1227–1249. 10.2147/IJN.S121956 (2017). 28243086 10.2147/IJN.S121956 PMC5317269

[36] Benelli, G. Mode of action of nanoparticles against insects. *Environ. Sci. Pollut. Res.* **25**(13), 12329–12341 (2018). 10.1007/s11356-018-1850-4 29611126

[37] Mehlhorn, H. (Ed) Nanoparticles in the fight against parasites. Parasitol. Res. Monographs vol. 8, Springer, Berlin, New York (2016).

[38] Elieh-Ali-Komi, D. & Hamblin, M. R. Chitin and Chitosan: production and application of versatile biomedical nanomaterials. *Int. J. Adv. Res. (Indore).* **4**(3), 411–427 (2016). 27819009 PMC5094803

[39] Shah, B. R. & Mraz, J. Advances in nanotechnology for sustainable aquaculture and fisheries. *Rev. Aquac*10.1111/raq.12356 (2019).

[40] Nangmenyi, G., & Economy, J. Nanometallic particles for oligodynamic microbialdisinfection. A. Street, R. Sustich, J. Duncan, N. Savage (Eds.) Nanotechnology applications for clean water: Solutions for improving water quality. William Andrew, Norwich, 283–295 (2014).

[41] Mostafa, N. A., Hamdi, S. A. H. & Fol, M. F. Potential anthelmintic effect of chitosan on *Syphacia muris* infecting Wistar rats: biochemical, immunological, and histopathological studies. *Sci. Rep.* **14**, 2825. 10.1038/s41598-024-52309-8 (2024). 38310115 10.1038/s41598-024-52309-8 PMC10838320

[42] Abdel-Latif, M., El-Shahawi, G., Aboelhadid, S. M. & Abdel-Tawab, H. Immunoprotective effect of Chitosan Particles on Hymenolepis nana infected mice. *Exp. Immunol.*10.1111/sji.12568 (2017). 10.1111/sji.12568 28513991

[43] Salem, H. et al. Morphological and molecular characterization of Ascaridia columbae in the domestic pigeon (Columba livia domestica) and the assessment of its immunological responses. *Poultry Sci.*. **101**(2), ISSN 0032–5791. 10.1016/j.psj.2021.101596 (2022). 10.1016/j.psj.2021.101596 PMC8693010 34929441

[44] Abdelsalam, M. et al. Investigating dynamics, etiology,pathology, and therapeutic interventions of Caligus clemensi and Vibrio alginolyticus co-infection in farmed marinefish. *Sci. Rep.* **14**(1), 20704 (2024). 10.1038/s41598-024-70528-x PMC11377424 39237535

[45] Abu-Elala, N. M., Attia, M. M. & Abd-Elsalam, R. M. Chitosan-silver nanocomposites in goldfish aquaria: a new perspective in *Lernaea cyprinacea* control. *Int. J. Biol. Macromol.* **111**, 614–622. 10.1016/j.ijbiomac.2017.12.133 (2018). 29292144 10.1016/j.ijbiomac.2017.12.133

